# Derivation and validation of a nomogram based on clinical characteristics to diagnose endometriosis associated ovarian cancer preoperatively

**DOI:** 10.1007/s00432-023-05524-1

**Published:** 2024-01-19

**Authors:** Ting Xu, Xianglin Nie, Lin Zhang, Huangyang Meng, Yi Jiang, Yicong Wan, Wenjun Cheng

**Affiliations:** Department of Gynecology, The First Affiliated Hospital of Nanjing Medical University, No. 300 Guangzhou Road, Nanjing, 210029 Jiangsu China

**Keywords:** Endometriosis associated ovarian cancer, Nomogram, Endometriosis, Diagnosis

## Abstract

**Purpose:**

The preoperative diagnosis of endometriosis associated ovarian cancer (EAOC) remains challenging for lack of effective diagnostic biomarker. We aimed to study clinical characteristics and develop a nomogram for diagnosing EAOC before surgery.

**Methods:**

A total of 87 patients with EAOC and 348 patients with ovarian endometrioma (OEM) were enrolled in our study. Least absolute shrinkage and selection operator (LASSO) regression and Logistic regression were utilized to select variables and construct the prediction model. The performance of the model was assessed using receiver operating characteristic (ROC) analyses and calibration plots, while decision curve analyses (DCAs) were conducted to assess clinical value. Bootstrap resampling was used to evaluated the stability of the model in the derivation set.

**Results:**

The EAOC patients were older compared to the OEM patients (46.41 ± 9.62 vs. 36.49 ± 8.09 year, P < 0.001) and proportion of postmenopausal women was higher in EAOC group than in the OEM group (34.5 vs. 1.5%, P < 0.001). Our prediction model, which included age at diagnosis, tumor size, cancer antigen (CA) 19–9 and risk of ovarian malignancy algorithm (ROMA), demonstrated an area under the curve (AUC) of 0.858 (95% confidence interval (CI): 0.795–0.920) in the derivation set (N = 304) and an AUC of 0.870 (95% CI: 0.779–0.961) in the validation set (N = 131). The model fitted both the derivation (Hosmer–Lemeshow test (HL) chi-square = 12.600, P = 0.247) and the validation (HL chi-square = 8.210, P = 0.608) sets well.

**Conclusion:**

Compared to patients with OEM, those with EAOC exhibited distinct clinical characteristics. Our four-variable prediction model demonstrated excellent performance in both the derivation and validation sets, suggesting its potential to assist with preoperative diagnosis of EAOC.

**Supplementary Information:**

The online version contains supplementary material available at 10.1007/s00432-023-05524-1.

## Introduction

Endometriosis (EM) is one of the most common gynecological diseases, and affects approximately 190 million women worldwide (Zondervan et al. 2020). Ovarian endometrioma (OEM) is one of the subtypes of EM along with superficial peritoneal endometriosis and deep infiltrating endometriosis (Horne and Missmer 2022). OEM is more common in premenopausal women than in postmenopausal women (Gorp et al. 2004), and over 50% of patients with OEM had symptoms such as pelvic pain in their adolescences (Becker et al. 2022). Malignancy was the most fatal comorbidity for patients with OEM and OEM had a 0.9–4.5% probability of malignant transformation to epithelial ovarian cancer (EOC) (Gorp et al. 2004), that is, endometriosis associated ovarian cancer (EAOC).

Compared to OEM, EAOC showed no obvious symptoms (Lheureux et al. 2019), so identifying the occurrence of EAOC in OEM patients during long-term follow-up was crucial. Researchers found clinical characteristics of EAOC differed from those of OEM, such as age at diagnosis older, tumor larger and elevated levels of serum tumor markers (Hermens et al. 2020; Younis and Izhaki 2023; Murakami et al. 2020). We found that patients with EAOC had elevated serum cancer antigen (CA) 19–9 and human epididymis protein 4 (HE4) in a previous study (Xu et al. 2023).

In this study, we enrolled a larger sample size and involved more clinical characteristics extensively. Employing least absolute shrinkage and selection operator (LASSO) regression and Logistic regression, we derived a nomogram to diagnose EAOC.

## Methods

### Study design

Our study was a retrospective, single centered, case control study based on electronic medical record system of our institution. The study had been approved by Institutional Review Board (Ethnics Committee of First Affiliated Hospital of Nanjing Medical University, date: 2020-10-12, ID: 2020-MD-371), while written informed consents were exempted for retrospective study.

### Study patients

**Fig. 1:**
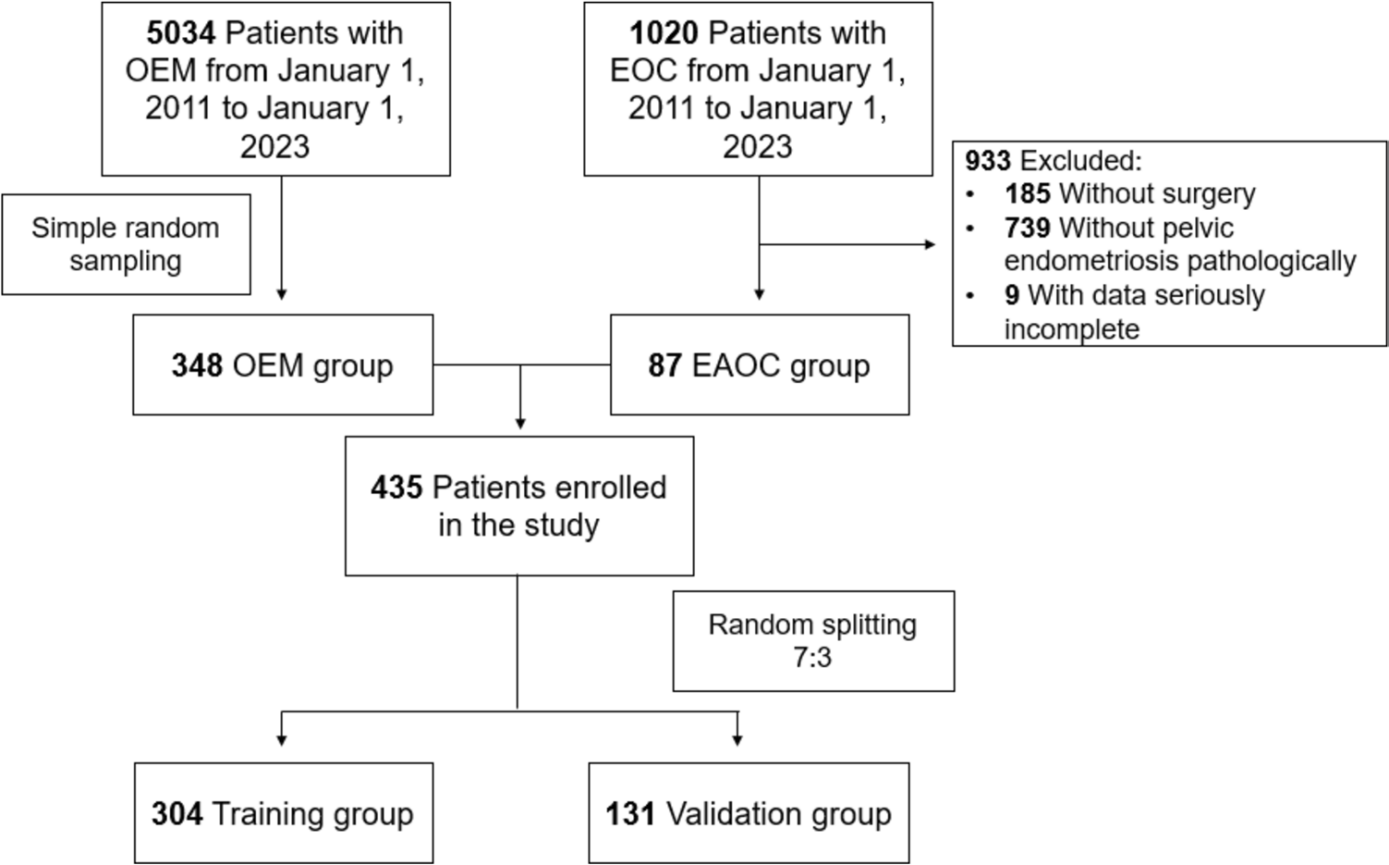
Flowchart of enrollment of study patients

Among 1020 patients diagnosed with EOC from January 1, 2011 to January 1, 2023 in our institution, we selected patients in accord with diagnostic standard of EAOC. Classical diagnostic standard of EAOC was first proposed by Sampson (1925) and later supplemented by Scott (1953). Firstly, cancer and EM coexisted in the same ovary. Secondly, the cancer arose from EM other than another site. Thirdly, glands and surrounding stroma coexisted in EM. Fourthly, morphological continuity was found between cancer and EM. However, more and more researchers found this diagnostic standard might be too strict to cause missed diagnosis (Younis and Izhaki 2023; Murakami et al. 2020; Kawahara et al. 2022; Zhu et al. 2021; Kawahara et al. 2021; Similä-Maarala et al. 2022; Chao et al. 2022; Hernández et al. 2022). According to Gorp et al. (2004), diagnostic standard of EAOC in this study was EOC with pelvic EM. We expelled 185 patients for without surgeries, 739 patients without pelvic EM proven by pathologic examination, and 9 with dominant clinical characteristics incomplete. Finally, 87 patients with EAOC were enrolled in study group (Fig. 1).

The control group of the study was selected from patients diagnosed with OEM in the same period as EAOC patients in our institution. To compensating for limitation of sample size of EAOC group, we enlarged fourfold the OEM group to 348 cases randomly selected from 5034 patients with OEM. A total of 435 patients composed the study population, and 70% of the patients (N = 304) were randomly sampled as derivation set, while the remainders (N = 131) were involved in validation set (Fig. 1).

### Data collection

Clinical characteristics of the patients were collected from their electronic medical records. Preoperative examinations were carried out within one week before surgeries. These characteristics covered demographics (age at diagnosis and body mass index (BMI)), reproductive and medical history (gravidity, parity, tumor size, previous abdominal surgery, hypertension, diabetes, other malignancy, uterine leiomyoma and other benign ovarian tumor), menstruation (dysmenorrhea and menopausal status), serologic tumor markers, white blood cell count and classification, coagulation function and blood biochemistry examination. Risk of ovarian malignancy algorithm (ROMA), deriving from CA125, HE4 and menopausal status, was proposed as a model assisting diagnosis of epithelial ovarian cancer (Gentry-Maharaj et al. 2020; Suri et al. 2021). Copenhagen index (CPH-I), calculated with age, CA125 and HE4 was another diagnostic model for indicating malignancy of adnexal masses (Karlsen et al. 2015). As inflammatory composite markers, monocyte lymphocyte ratio (MLR) and neutrophil lymphocyte ratio (NLR) were used to study diagnosis and prognosis of OC (Zhang et al. 2023; Leng et al. 2022). Thus, we calculated above indicators to analyze clinical characteristics of these patients comprehensively.

### Statistical analysis

Continuous variates fitting and not fitting normal distribution were described as means with standard deviations and medians with quartile ranges, respectively, while categorical variables were described as counts and proportions. To comparing differences between clinical characteristics of patients with EAOC and OEM, t-tests were used for normally distributed continuous variates, while rank sum tests were used for non-normally distributed continuous variates and rank variates. Chi-square tests or Fisher’s exact tests were used for categorical variables.

LASSO regression was employed to select variables included in prediction model, and multivariate Logistic regression were used to build model. To examine stability of model in derivation set, a bootstrap resampling with 1000 repetitions was performed. Receiver operating characteristic analyses (ROCs) and areas under curves (AUCs) were used to estimate discrimination sensitivities of the model, while calibration curves were employed to evaluate comparisons between predicted and actual diagnoses in derivation and validation sets. Decision curve analyses (DCAs) were performed to show clinical benefit of the model. The model was visualized as nomogram and web-based dynamic nomogram to be easily used in clinical practice.

Statistical analyses were performed using R software version 4.1.2 and Stata SE version 15. All statistical comparisons were two-sided, and differences were significant at P < 0.05.

## Results

### Clinical characteristics of patients with EAOC and OEM

Compared to the OEM group, patients in the EAOC group were older (EAOC vs. OEM: 46.41 ± 9.62 vs. 36.49 ± 8.09 year, P < 0.001) and had higher BMIs (23.45 ± 2.97 vs. 22.01 ± 3.18 kg/m2, P < 0.001). Additionally, patients with EAOC had higher times of pregnancies (2 (1–3) vs. 1 (0–1), P = 0.002) and deliveries (1 (1–1) vs. 1 (0–1), P < 0.001) compared to patients with OEM. Furthermore, EAOC patients were more likely to be postmenopausal women (30 (34.5%) vs. 5 (1.4%), P < 0.001) and had higher proportions of individuals with hypertension (13 (14.9%) vs. 10 (2.9%), P < 0.001), diabetes (3 (3.4%) vs. 2 (0.6%), P = 0.092) and other malignancies (8 (9.2%) vs. 15 (4.3%), P = 0.120). Conversely, a higher proportion of OEM patients experienced dysmenorrhea (25 (28.7%) vs. 218 (62.6%), P < 0.001), and the detection rates of uterine leiomyoma (11 (12.6%) vs. 135 (38.8%), P < 0.001) and other ovarian benign tumors (1 (1.1%) vs. 29 (8.3%), P = 0.018) were also higher in this group (Table 1).

**Table 1:** Clinical characteristics of patients in EAOC group and OEM group

Variate	EAOC group N = 87	OEM group N = 348	*P* value
Age at diagnosis (year)	46.41 ± 9.62	36.49 ± 8.09	< 0.001
BMI (kg/m^2^)	23.45 ± 2.97	22.01 ± 3.18	< 0.001
Gravidity	2 (1–3)	1 (0–1)	0.002
Parity	1 (1–1)	1 (0–1)	< 0.001
Tumor size (cm)	9.44 ± 4.45	6.55 ± 4.20	< 0.001
Dysmenorrhea (%)	25 (28.7)	218 (62.6)	< 0.001
Postmenopausal (%)	30 (34.5)	5 (1.4)	< 0.001
Previous abdominal surgery (%)	37 (42.5)	153 (44)	0.809
Hypertension (%)	13 (14.9)	10 (2.9)	< 0.001
Diabetes (%)	3 (3.4)	2 (0.6)	0.092
Other malignancy (%)	8 (9.2)	15 (4.3)	0.120
Uterine leiomyoma (%)	11 (12.6)	135 (38.8)	< 0.001
Other benign ovarian tumor (%)	1 (1.1)	29 (8.3)	0.018
CA125 (U/mL)	78.29 (25.19–207.60)	50.45 (30.73–82.25)	0.004
CA19-9 (U/mL)	35.45 (13.75–143.21)	13.87 (2.95–27.77)	< 0.001
HE4 (pmol/L)	67.17 (54.74–99.64)	49.35 (41.53–58.45)	< 0.001
ROMA (%)	15.94 (10.64–47.63)	7.77 (5.30–11.29)	< 0.001
CPH-I	− 3.71 (− 4.38–− 3.05)	− 2.80 (− 3.65–− 1.34)	< 0.001
WBC (× 10^9^/L)	6.57 ± 2.15	5.93 ± 2.52	0.017
NR	0.65 ± 0.09	0.58 ± 0.13	< 0.001
LR	0.27 ± 0.08	0.34 ± 0.09	< 0.001
MLR	0.23 (0.17–0.30)	0.17 (0.14–0.22)	< 0.001
NLR	2.43 (1.75–3.33)	1.69 (1.24–2.23)	< 0.001
DD2 (mg/L)	0.40 (0.25–1.06)	0.24 (0.16–0.33)	< 0.001
FIB (g/L)	2.81 (2.42–3.61)	2.42 (2.11–2.74)	< 0.001
HDL	1.18 (0.24)	1.31 (0.27)	0.001
LDL	2.96 (0.71)	2.67 (0.68)	0.005
TP	66.85 (4.66)	67.48 (5.69)	0.380
AGR	1.47 (1.32–1.56)	1.53 (1.37–1.69)	0.012
Histological type			
Clear cell carcinoma	46 (52.9)	–	–
Endometrioid carcinoma	31 (35.6)	–	–
Serous carcinoma	7 (8.0)	–	–
Mucinous carcinoma	3 (3.4)	–	–

*BMI* body mass index, *CA* cancer antigen, *HE4* human epididymis protein 4, *ROMA* risk of ovarian malignancy algorithm, *CPH-I* Copenhagen index, *WBC* white blood cell, *NR* neutrophil ratio, *LR* lymphocyte ratio, *MLR* monocyte lymphocyte ratio, *NLR* neutrophil lymphocyte ratio, *DD2* d dimer, *FIB* fibrinogen, *HDL* high-density lipoprotein, *LDL* low-density lipoprotein, *TP* serum total protein, *AGR* albumin globulin ratio

Further analysis revealed distinct differences in the results of preoperative blood tests between the patients in the two groups. Patients with EAOC exhibited higher levels of CA125, CA19-9, HE4, and ROMA compared to patients with OEM. Additionally, these patients had elevated levels of white blood cell counts and higher percentages of neutrophils, while their percentages of lymphocytes were lower, resulting in higher MLRs and NLRs. Moreover, the EAOC group showed higher levels of fibrinogen (FIB) and d-dimer (DD2), which suggests more severe states of hypercoagulability. Contrastingly, patients with EAOC had lower levels of high-density lipoprotein (HDL) and low-density lipoprotein (LDL), as well as lower levels of total protein (TP) and albumin globulin ratio (AGR), indicating poorer nutritional statuses (Table 1).

Among 87 patients with EAOC, histological type of 46 (52.9%) patients was clear cell carcinoma, that of 31 (35.6%) patients was endometrioid carcinoma, that of seven (8.0%) patients was serous carcinoma and that of three (3.4%) patients was mucinous carcinoma (Table 1).

### Development of prediction model

**Fig. 2:**
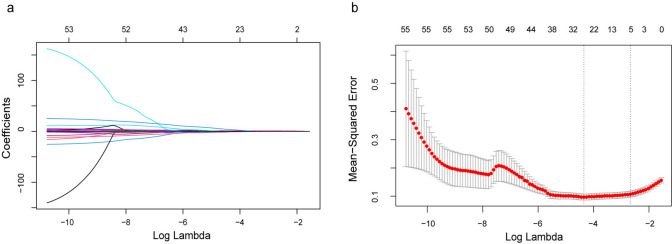
LASSO regression analysis of clinical characteristics of patients in derivation set. **a** LASSO regression. **b** Tenfold cross validation

The derivation set consisted of 59 patients with EAOC and 245 patients with OEM, and the characteristics of 304 patients were used to construct the prediction model. LASSO regression was used to screen variables, and Logistic regression was used to construct the model. All variables were entered into LASSO regression with tenfold cross-validation, and at the lambda value equal to 0.06882337, variables with nonzero coefficients, including age, postmenopausal, tumor size, CA19-9 and ROMA (Fig. 2).

Since menopausal status played a role in calculating ROMA, to minimize the interaction effect, we excluded postmenopausal from construction of the model. Finally, a four-variable prediction model was constructed using age, tumor size, CA19-9 and ROMA. Older age, larger tumor size, higher CA19-9 and higher ROMA were identified as independent risk factors (Table 2).

**Table 2:** The four-variate prediction model for diagnosing EAOC

Variate	OR	OR 95% CI	*P* value
Age at diagnosis (year)	1.105	1.056–1.161	< 0.001
Tumor size (cm)	1.176	1.066–1.309	0.002
CA19-9 (U/mL)	1.003	1.000–1.007	0.042
ROMA (%)	1.071	1.039–1.114	< 0.001

The model was constructed with clinical characteristics of patients in derivation set via multivariate logistic regression

*CA* cancer antigen, *ROMA* risk of ovarian malignancy algorithm

### Performance of prediction model

**Fig. 3:**
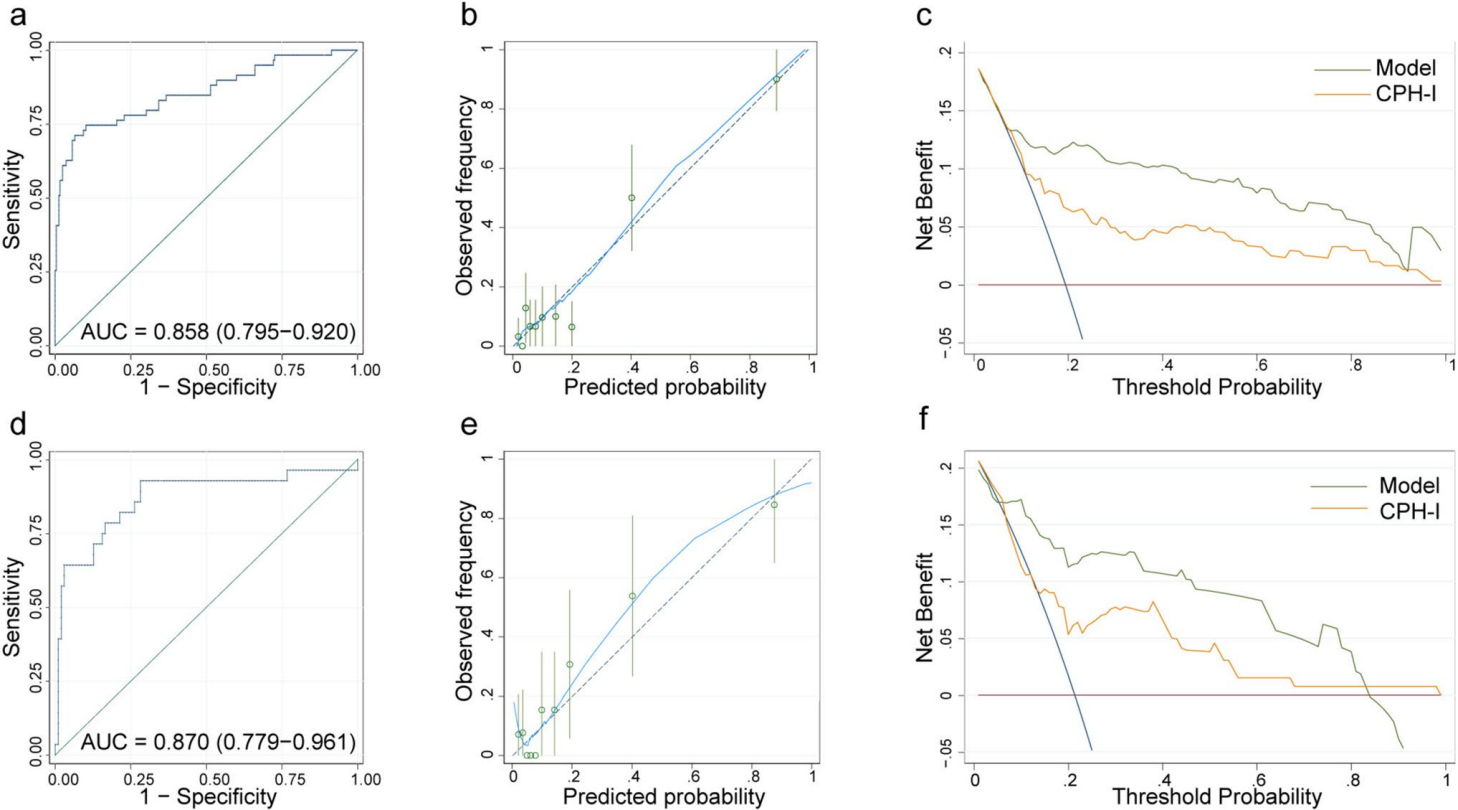
Performance of prediction model in derivation set and validation set. **a** ROC curve of derivation set. **b** ROC curve of validation set. **c** Calibration curve of derivation set. **d** Calibration curve of validation set. **e** DCA curve of derivation set. **f** DCA curve of validation set

The performance of the predictive model was assessed in both the derivation and validation sets. ROC analysis and calibration curves were employed to evaluate the accuracy and agreement of the model in diagnosing EAOC, while DCA was utilized to assess the clinical utility of the model. In the training set, the AUC value for diagnosing EAOC was 0.858 (95% CI 0.795–0.920) (Fig. 3). The adjusted AUC value, obtained through 1000 repeated bootstrap resampling, was also 0.855 (95% CI 0.788–0.916), showing good consistency in the derivation set. The calibration curve demonstrated a good fit of the model in the training set (Hosmer–Lemeshow (HL) test chi-square = 12.600, P = 0.247). According to the DCA curve, this model exhibited superior clinical benefits over CPH-I within the threshold range of 0.08–0.92. Furthermore, our model showed superior performance compared to CPH-I in diagnosing EAOC, as evidenced by a higher AUC value (EAOC vs. OEM: 0.858 vs. 0.746, Delong test Z = 3.093, P = 0.002) and more favorable sensitivity (71.19% vs. 55.93%) and specificity (93.47% vs. 82.04%) (Table S2). Similarly, in the validation set, the ROC curve showed an AUC value of 0.870 (95% CI: 0.779–0.961) for the model's diagnostic performance in EAOC. The calibration curve indicated a good fit of the model in the training set (HL test chi-square = 8.210, P = 0.608), and the DCA curve demonstrated that the threshold range for clinical benefit was between 0.07 and 0.83, favoring this model over CPH-I (Fig. 3).

### Visualization of prediction model

**Fig. 4:**
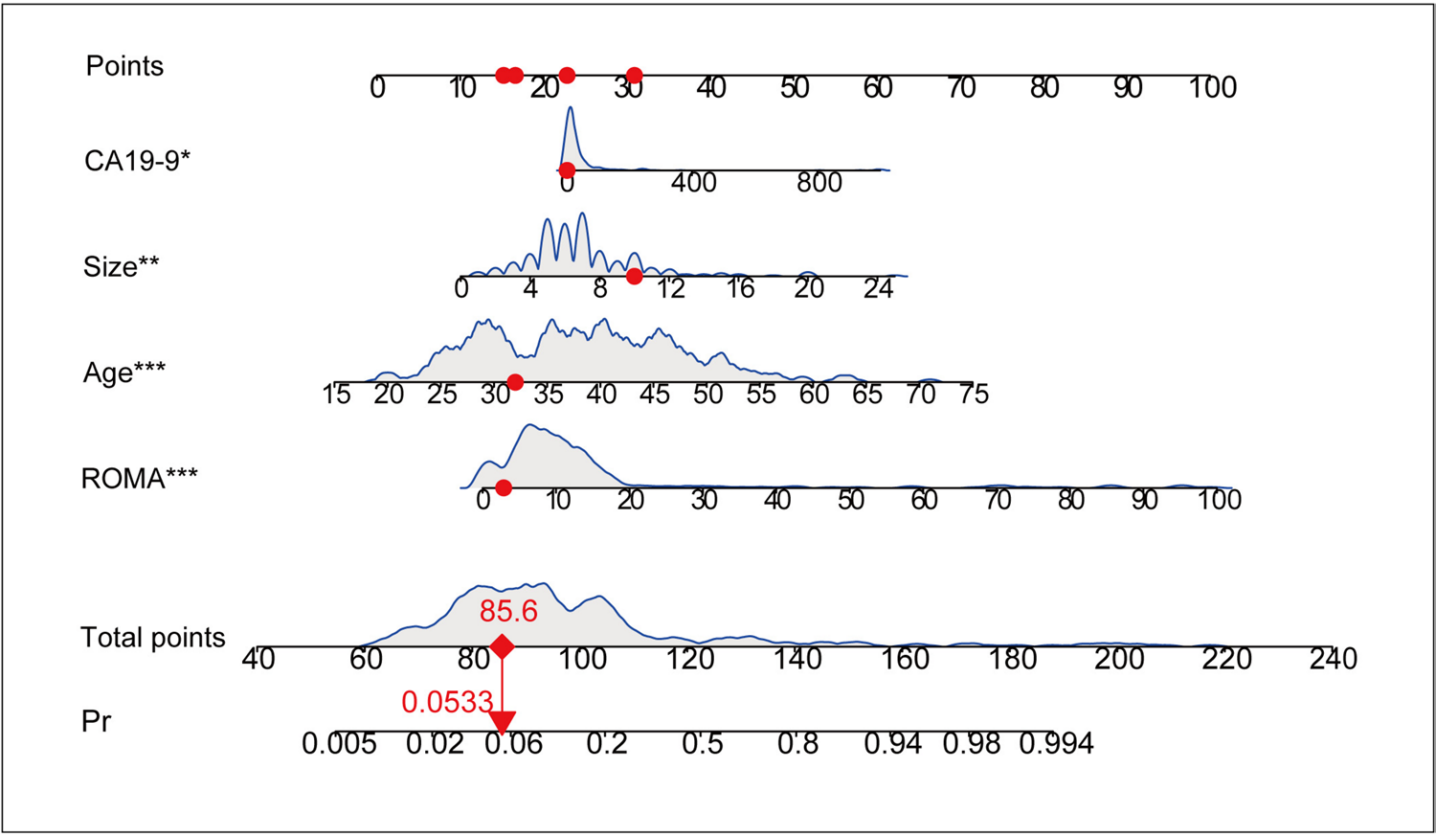
Nomogram of four-variate prediction model

To facilitate the practical application of the predictive model, we presented it in the form of a nomogram (Fig. 4) and a web-based dynamic nomogram (https://tingxu-1.shinyapps.io/dynnomapp/). For instance, let’s consider the first patient in the validation set, the patient's age of 32 years corresponded to 16.8 points, lesion size of 10 cm corresponded to 30.6 points, CA19-9 level of 1.99 corresponded to 22.4 points, ROMA value of 2.88 corresponded to 15.8 points, resulting in a total score of 85.6 points. Consequently, the probability of diagnosing EAOC for this patient was calculated as 0.0533, and the actual diagnosis was OEM.

## Discussion

EAOC is a unique type of EOC that arises from endometriosis. OEM is a benign disease with high prevalence and recurrence rates (Allaire et al. 2023), and was often diagnosed at a young age (Nnoaham et al. 2011). Consequently, a large number of patients with OEM required long-term managements, making EAOC a rare but life-threatening complication that affected these women. As a subtype of EOC, EAOC mostly lacked typical symptoms and was hard to diagnose before surgery (Younis and Izhaki 2023). In this study, we found that the proportion of EAOC patients with symptoms such as dysmenorrhea was lower compared to those with OEM. Therefore, an effective and convenient method for early diagnosis of EAOC is an urgent issue in the long-term management of OEM.

Since the first report by Sampson (1925), extensive research has been conducted worldwide to investigate and understand special features of EAOC. Through a systematic review of previous study, Younis et al. found that 70% of patients diagnosed with EAOC were over the age of 50, and a tumor size larger than 9 cm was identified as a risk factor for EAOC (Younis and Izhaki 2023). Phung et al. (2022) revealed that EAOC patients might have a higher BMI compared to patients with OEM. Chao et al. (2022) discovered that the significance of CA125 and CA19-9 in diagnosing EAOC. Our previous study found that age at diagnosis over 42 years, tumor size over 9.2 cm, and elevated CA19-9 and HE4 levels are risk factors for EAOC (Xu et al. 2023). In this study, we expanded the population and further validated these findings.

EAOC exhibited distinct clinical characteristics compared to OEM, highlighting the need for a portable and effective preoperative diagnostic model using machine learning. Chao et al. (2022) constructed a diagnostic model for EAOC using gradient decision trees and demonstrated its favorable performance. However, the drawback of this model was its reliance on a computer, which made it inconvenient and limited its widespread usage. To address this limitation, our study employed LASSO regression to select variables and Logistic regression to construct the model. The model was presented and utilized in the form of nomogram, offering ease of use and potential assistance to clinicians in diagnosing EAOC. Our prediction model incorporated age, tumor size, CA19-9, and ROMA as variates. Furthermore, we assessed the model's performance in both the training and validation sets using ROC analysis, calibration curves, and DCA, and found that it performed well.

Our study successfully developed an easy-to-use model for preoperative diagnosis of EAOC. Additionally, we employed machine learning-based LASSO regression and Logistic regression to reduce confounding bias in this retrospective observational study. To minimize selection bias, simple random sampling was used in selecting control groups. However, the study had limitations, including a small sample size and the presence of unavoidable information bias. Additionally, absence of an external validation cohort could limit our prediction model widely used. In order to address these limitations, we plan to collaborate with domestic and international research groups for further study.

In this study, we studied clinical characteristics of patients with EAOC and developed a preoperative diagnostic model that incorporated age, tumor size, CA19-9, and ROMA. We found that this model has a favorable performance in our cohort and was potential to aid clinicians in early diagnosis of EAOC.

### Supplementary Information

Below is the link to the electronic supplementary material.

Supplementary file1 (DOCX 17 KB)

## Data Availability

The datasets generated during and/or analysed during the current study are available from the corresponding author on reasonable request.
