# Supplementary material for: Derivation and validation of a nomogram based on clinical characteristics to diagnose endometriosis associated ovarian cancer preoperatively
Source: J Cancer Res Clin Oncol. 2024 Jan 19;150(1):19. doi: 10.1007/s00432-023-05524-1 (PMC10799100; doi:10.1007/s00432-023-05524-1)
Supplement: Supplementary file 1 — Supplementary file1 (DOCX 17 KB) [file 432_2023_5524_MOESM1_ESM.docx]

Table S1. Clinical characteristics of patients in derivation and validation sets.

| Variate | Derivation set  N = 304 | Validation set  N = 131 | *P* value |
| --- | --- | --- | --- |
| Patients with EAOC (%) | 59 (19.4) | 28 (32.2) | 0.221 |
| Age at diagnosis (year) | 38.29 ± 9.31 | 38,92 ± 9.31 | 0.518 |
| BMI (kg/m^2^) | 22.40 ± 3.31 | 22.05 ± 2.87 | 0.265 |
| Gravidity | 1 (1–2) | 1 (1–2) | 0.434 |
| Parity | 1 (0–1) | 1 (0–1) | 0.745 |
| Tumor size (cm) | 6.99 ± 3.28 | 6.98 ± 2.99 | 0.969 |
| Dysmenorrhea (%) | 170 (55.9) | 73 (55.7) | 0.970 |
| Postmenopausal (%) | 24 (7.9) | 11 (8.4) | 0.860 |
| Previous abdominal surgery (%) | 139 (45.7) | 51 (38.9) | 0.190 |
| Hypertension (%) | 17 (5.6) | 6 (4.6) | 0.665 |
| Diabetes (%) | 4 (1.3) | 1 (0.8) | 0.996 |
| Other malignancy (%) | 14 (4.6) | 9 (6.9) | 0.333 |
| Uterine leiomyoma (%) | 98 (32.2) | 48 (36.6) | 0.428 |
| Other ovarian benign tumor (%) | 22 (7.2) | 8 (6.1) | 0.684 |
| CA125 (U/mL) | 51.05 (30.40–84.93) | 56.40 (31.90–115.20) | 0.224 |
| CA19-9 (U/mL) | 17.30 (5.52–34.75) | 514.61 (4.60–35.91) | 0.863 |
| HE4 (pmol/L) | 52.90 (42.71–63.31) | 51.40 (41.83–60.64) | 0.403 |
| ROMA (%) | 9.19 (5.69–13.37) | 8.73 (5.77–12.52) | 0.534 |
| CPH-I | -3.60 (-4.18–-2.80) | -3.64 (-4.25–-2.86) | 0.527 |
| WBC (*10^9^/L) | 6.13 ± 2.76 | 5.89 ± 1.58 | 0.355 |
| NR | 0.59 ± 0.11 | 0.60 ± 0.15 | 0.561 |
| LR | 0.33 ± 0.09 | 0.33 ± 0.09 | 0.963 |
| MLR | 0.19 (0.14–0.24) | 0.18 (0.14–0.23) | 0.389 |
| NLR | 1.76 (1.28–2.43) | 1.81 (1.39–2.47) | 0.769 |
| DD2 (mg/L) | 0.25 (0.16–0.37) | 0.27 (0.17–0.42) | 0.150 |
| FIB (g/L) | 2.44 (2.14–2.82) | 2.56 (2.18–2.94) | 0.171 |
| AGR | 1.52 (1.38–1.69) | 1.47 (1.35–1.63) | 0.122 |

Abbreviations: BMI, body mass index; CA, cancer antigen; HE4, Human epididymis protein 4; ROMA, risk of ovarian malignancy algorithm; CPH-I, Copenhagen index; WBC, white blood cell count; NR, neutrophil ratio; LR, lymphocyte ratio; MLR, monocyte lymphocyte ratio; NLR, neutrophil lymphocyte ratio; DD2; d dimer; FIB, fibrinogen; AGR, albumin globulin ratio.

Table S2. Diagnostic performance for four-variate prediction model compared to CPH-I in derivation set.

| Model | AUC | AUC 95% CI | Sensitivity (%) | Specificity (%) |
| --- | --- | --- | --- | --- |
| Four-variate model | 0.858 | 0.795–0.920 | 71.19 | 93.47 |
| CPH-I | 0.746 | 0.672–0.820 | 55.93 | 82.04 |

Abbreviations: CPH-I, Copenhagen index.
